# Tissue Regeneration: A Silk Road

**DOI:** 10.3390/jfb7030022

**Published:** 2016-08-05

**Authors:** Dave Jao, Xiaoyang Mou, Xiao Hu

**Affiliations:** 1 Department of Physics and Astronomy, Rowan University, Glassboro, NJ 08028, USA; jaod07@students.rowan.edu; 2 Department of Biomedical Engineering, Rowan University, Glassboro, NJ 08028, USA; 3 Department of Chemistry and Biochemistry, Rowan University, Glassboro, NJ 08028, USA; hu@rowan.edu; 4 Department of Biomedical and Translational Sciences, Rowan University, Glassboro, NJ 08028, USA

**Keywords:** silk fibroin, tissue engineering, artificial bone, eye, nerve, skin, tendon, ligament, cartilage

## Abstract

Silk proteins are natural biopolymers that have extensive structural possibilities for chemical and mechanical modifications to facilitate novel properties, functions, and applications in the biomedical field. The versatile processability of silk fibroins (SF) into different forms such as gels, films, foams, membranes, scaffolds, and nanofibers makes it appealing in a variety of applications that require mechanically superior, biocompatible, biodegradable, and functionalizable biomaterials. There is no doubt that nature is the world’s best biological engineer, with simple, exquisite but powerful designs that have inspired novel technologies. By understanding the surface interaction of silk materials with living cells, unique characteristics can be implemented through structural modifications, such as controllable wettability, high-strength adhesiveness, and reflectivity properties, suggesting its potential suitability for surgical, optical, and other biomedical applications. All of the interesting features of SF, such as tunable biodegradation, anti-bacterial properties, and mechanical properties combined with potential self-healing modifications, make it ideal for future tissue engineering applications. In this review, we first demonstrate the current understanding of the structures and mechanical properties of SF and the various functionalizations of SF matrices through chemical and physical manipulations. Then the diverse applications of SF architectures and scaffolds for different regenerative medicine will be discussed in detail, including their current applications in bone, eye, nerve, skin, tendon, ligament, and cartilage regeneration.

## 1. Introduction

Silk fibroins (SF) are a class of natural protein with the ability to be extremely bioactive. In terms of availability, the domesticated mulberry *Bombyx mori* (*B. mori*) silkworms are the main producers of silk derived from the large-scale cultivation of silkworms through sericulture [1]. Mainly cultivated in plantations for high-end textile fibers, the application potential of SF is multidimensional, such as in biotechnology, material science, medicine, and pharmaceuticals [2]. SF have also found uses in optics and photonics, electronics, and optoelectronic applications [3]. The multiple functionalities of SF offer a range of modifications for preparing a wide spectrum of derivatives for biomedical applications. Also, alternative choices to *B. mori* silk are the non-mulberry tropical silkworm silks such as tasar (*Antheraea mylitta*) silk, which possesses higher mechanical strength and better cell adhesion due to the presence of Arg-Gly-Asp (RGD) recognition sequences [1]. With enhanced cell attachment and tunable degradation, non-mulberry silk fibroins can be employed in various forms of biomaterials, such as SF microparticles for drug delivery vehicles or microcarriers using wet-milling and spray drying techniques [4]. The biomedical and therapeutic significance of SF derivatives and matrices have been the subject of extensive study over the past several years. For example, with *Antheraea mylitta* silk matrices, 3D in vitro tumor model systems can be constructed to mimic in vivo microenvironments to show different zones of cancer cell proliferation in avascular tumors [5]. Systems that resemble in vivo situations can be effective in evaluating and screening new drug efficiency and strategies while decreasing the use of experimental animals [5]. 

**Figure 1:**
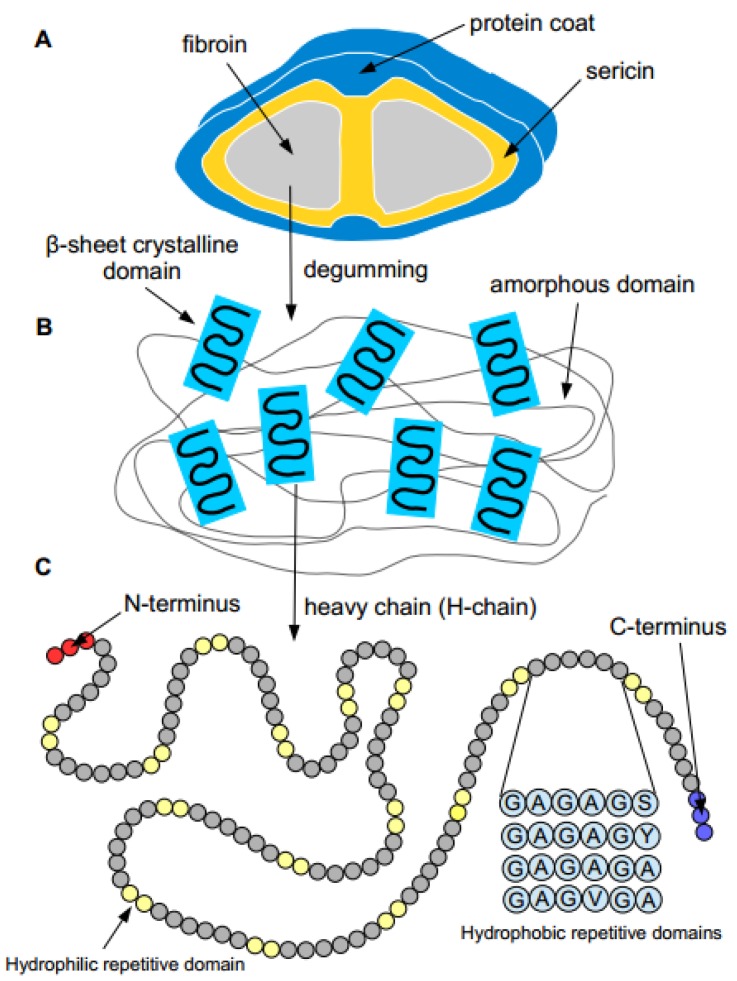
A schematic illustration of silk fibers produced by silkworms. (**A**) The raw silk fiber is composed of two fibroin fibers held together with sericin covered with a protein coat. After degumming, the removal of sericin, the fibroin fibers are dissolved in solution; (**B**) The illustration of β-sheet crystallite embedded in the amorphous matrix of silk fibroin fibers; (**C**) Each silk fibroin heavy chain (H-chain) consists of hydrophobic and hydrophilic repetitive domains. Each hydrophobic subdomain consists of different repeating units of hexapeptides [3].

Silk fibers are primarily composed of two proteins, fibroin and sericin, consisting of 18 different amino acids [2]. The amino acid sequence of SF, comprising of predominantly glycine, alanine, and serine (illustrated in Figure 1), can vary from species to species, which results in differences in chemical and mechanical properties. For instance, SF samples that contain a high amount of poly-Ala sequences have a more highly ordered crystalline structure and the repetitive stretches of poly-Ala also make it less soluble in acidic solvents, while SF samples containing poly-Gly-Ala sequences have mostly beta-sheet regions [1]. The mechanic strength and the insolubility of SF in common solvents, such as water, dilute acids and bases, and ethanol, are contributed by its unique sequence and physical formation [1]. Four different polymorphic forms, (anti)polarity and (anti)parallelity, have been proposed, with varying properties. They differ in their arrangement of polymeric chains, and among the polymeric strands, three have been suggested as being the crystal structure of SF [3]. The mechanical performance of SF is directly related to its molecular constituent and packing. Both the content and sequence of these amino acids will determine the physical and chemical properties of silk polymers.

**Figure 2:**
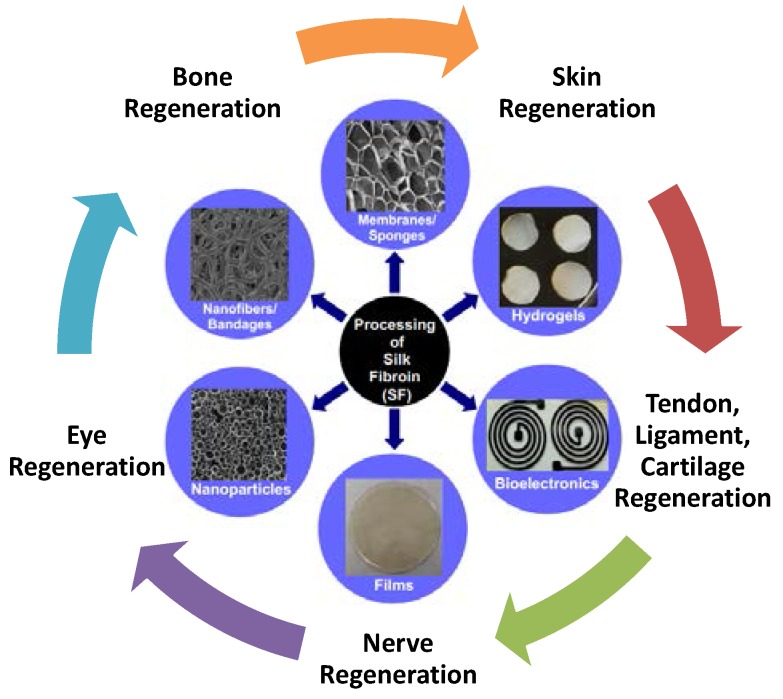
Schematic representation on the possibilities of processing silk fibroin (SF) into different forms with various biomedical applications.

Many reports have been published in previous years on the use of mulberry silks in a variety of applications, such as tissue engineering, wound healing, and drug delivery. With a natural healing biomaterial, the goal is to potentially replace current sutures and screws that can cause trauma when implanted into bone and tissue [2]. As a means to improve tendon repairs, novel knitted, non-mulberry silk fibroin scaffolds containing cell-binding RGD motifs have also been developed to replace the traditional polyethylene and polyester sutures [6]. With favorable interactions in biological systems and minimal immunological responses, SF materials have demonstrated good biocompatibility with various cell types by supporting and promoting adhesion, proliferation, growth, and differentiation of cells, leading to tissue regeneration. SF is easily processed into gels, membranes, nanofibers, films, nanoparticles, scaffolds, and foam-like forms (Figure 2), which makes it useful in the development of matrices/morphologies for the delivery of bioactive molecules, growth factors, signaling cues, and drug release profiles [3]. In this review, we discuss some of the recent findings on the potential application of SF materials and their functionalities from the mechanical and adherence perspectives. Also, the incorporation of SF in composite systems for tissue engineering, regenerative medicine, drug delivery, and wound healing will be discussed in detail.

## 2. Properties of Silk Proteins

Silk proteins can mainly be isolated from mulberry and non-mulberry silkworms from cocoons, and spiders occurring from their draglines. However, it is impractical to obtain large quantities of silk from spiders, while silkworms fibers are widely accepted and have found applications in biomedicine, bioengineering, and materials science [3]. In silk cocoons, the silk fibers consist of two parallel silk fibroin (SF) called brins, held together with a layer of sericin glue on the surface. Silk cocoons are composed of 60%–80% fibroin, 15%–35% sericin, and 1%–5% non-sericin component [7]. SF can be fabricated conventionally into films, hydrogels, and scaffolds when dissolved in solution [3]. The various morphologies of SF appear to possess several desirable and suitable properties: (i) improved biocompatibility with minimal immunogenicity, which is favorable in cell adhesion; (ii) ease of functionalities with topographic and morphological cues for various cellular interactions; and (iii) a tremendous diversity of mechanical architectures and structures, mimicking a variety of tissues ranging from soft to hard [2]. The preparation of SF materials from silk fiber requires a degumming process to remove the sericin. The post-extraction avoids acute and chronic inflammatory tissue reaction, which allows for successful implantation of scaffolds and cell culture [2]. Further processing steps through dissolution in aqueous or organic solvents permits the production of regenerated SF solution for easier processability and fabrication of silk-based biomaterials [3]. SF can be produced as a biomaterial in two primary ways: as a long-term biostable SF material or as a short-term biodegradable material, which can be controlled by the beta-sheet crystal content in the silk materials [2]. Through gelation processes, SF can be transformed into a three-dimensional hydrogel or sponge-like materials with potential applications in organ engineering. 

In recent years, many articles dealing with the applications of SF scaffolds have been published in tissue engineering. The versatility process of SF makes the fabrication of a wide range of functional architectures and morphologies possible. Functionalized surfaces, such as antibodies, dyes, drugs, fluorescent, growth factors, peptides, and nanoparticles, can be incorporated into SF-based three-dimensional constructs. Being that SF is a natural protein, the presence of the amino functionality make it easily modifiable to impart desired physical properties, such as electrical conductivity, optical properties and solubility, and distinctive biological functions, which can include antibacterial properties, cellular responses, and therapeutic effects [2]. With a high number of tyrosine residues in SF, a tyrosine-specific diazonium coupling reaction can be employed to change the protein surface chemistry and hydrophilicity to initiate mesenchymal stem cells’ (MSCs) attachment, proliferation, and differentiation in cell culture scaffolds [2]. Non-mulberry and mulberry SF scaffolds are suitable substrates for osteogenic and adipogenic differentiation of bone marrow cells (BMCs) expressing osteopontin (Spp1), osteocalcin (Bglap2), and osteonectin (Sparc) genes under osteogenic conditions and peroxisome proliferator activated receptor gamma (PPARg2), lipoprotein lipase (LPL), and adipocyte binding protein (aP2) genes under adipogenic conditions [8]. Also, the conjugation between fibroin and poly(D,L-lactic acid) or RGD can give rise to a higher degree of cell attachment and proliferation on substrates or scaffold [9]. Apart from the amino groups, SF can undergo many reactions such as addition, coupling, cross-linking, and graft copolymerization. 

An alternative approach to chemical modification is the blending of SF with other natural and synthetic polymers to incorporate different properties of interest. Variants of poly(ethylene glycol), poly(ε-caprolactone) (PCL), and poly(vinyl alcohol) can be incorporated into SF as reinforcement [10]. The incorporation of synthetic polymers can significantly improve the mechanical properties of SF, adding tunable porosity and a durable framework to the scaffold. Through blending, SF can be combined with the hard and stiff phases of natural or synthetic polymers, which can increase and decrease the elastic modulus and the compressive strength of the composite. Reports have shown that the added stiffness of PCL/silk scaffold composite can increase osteoblast attachment and differentiation on those of the untreated ceramic in bone tissue engineering [2]. To mimic natural bone, non-mulberry silk fibroin scaffolds grafted with polycaprolactone (PCL) nanofibers are fabricated using electrospinning technique and mineralized with calcium phosphate and nano-hydroxyapatite (nHAp) using electrodeposition. The mineralized non-mulberry silk fibroin scaffold replicates both the architecture and chemical composition of bones, enhancing the bioactivity, proliferation, and differentiation of osteoblast-like cells [11]. In other reports, the electrospinning of chitosan/SF biocomposites through a process solution or melt can also improve the physical stability over either chitosan or SF alone in wound dressings [12]. With the introduction of water-soluble chitosan into SF scaffolds, the biocomposite can form highly porous, water-stable scaffolds that can improve water retention, suitable for cell regeneration [13]. 

While SF is extensively studied and used in the biomedical and textile industry, silk sericin (the outer adhesive protein coating) is frequently abandoned and discharged as a byproduct or waste in the degumming process. Long dismissed as a strong immunological response trigger until recently, silk sericin has now been found to be a highly useful biomaterial with strong bioactivity and good cytocompatibility. Reports conclude that silk sericin can be a biocompatible biopolymer with very low immunogenicity, if not associated with fibroin fibers during the isolation and purification process [14]. Its promotion of keratinocytes and fibroblasts have led to the development of non-mulberry tasar cocoon silk sericin/polyacrylamide biomaterials for skin tissue repair, as a reconstructive dermal sealant [15]. Similarly, not only SF but silk sericin–PVA biomaterials can be prepared by cross-linking with genipin into films, scaffolds, and hydrogels tunable for bone, dermal, and neural tissue engineering [16]. For drug delivery, sericin encapsulated in alginate microparticles may also be employed in a rate-controlled manner, improving the bioactivity of conjugated drugs [17]. While current purifying procedures for sericin protein do not guarantee uniform in molecular size, composition, and biological activity, future advancements in extraction and recovery methods can lead to many cosmetic and biomedical applications [7]. 

In this review, we focus on the current parameters employed in the designing and fabricating of different silk-based structures to address and meet certain functions and specifications for cell outgrowth and drug delivery. Furthermore, we summarize and discuss the various strategies utilized to control and enhance the bio-chemical and mechanical properties of SF to ensure the functionality and durability of the material during cell differentiation and regeneration.

## 3. Silk Materials for Tissue Regenerations

### 3.1. Silk for Bone Regeneration

**Figure 3:**
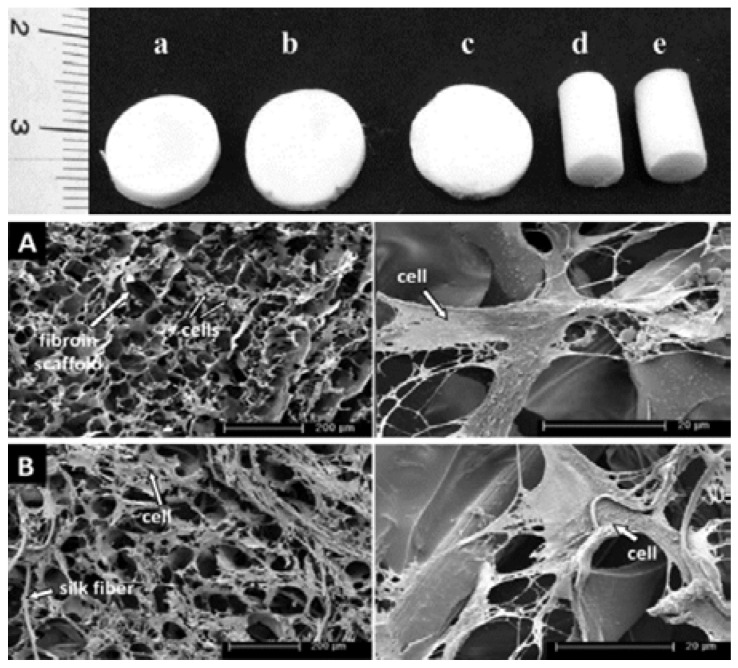
HMSC attachment and morphology on fibroin scaffolds (SF) and fiber/fibroin composite scaffolds (FRF), made of 4 wt % fibroin solution, after cultivation in culture medium for 21 days: (**A**) SF2; (**B**) FRF3, magnification 200 (left) and 3200 (right). (Reproduced with permission from Ref [20], Copyright Wiley Inc., 2013)

Bone tissue engineering is a promising strategy to regenerate natural bone through artificial means. Bone, a rigid organ, constitutes a major component of the human body. It serves numerous vital functions in the human body, including strong mechanical structure for support, dense and organized connective tissue for the protection of soft organs, and highly specialized cells for blood production and storage of minerals [18]. Bone is mainly comprised of collagen, the organic component, and carbonated hydroxyapatite (HA), the inorganic component, along with other growth factors and other non-collagenous proteins [18]. Like any tissue engineering approach, bone tissue engineering can be transplanted as grafts from other parts of the body or cadavers, or with metallic alloys, but these strategies have many disadvantages [18]. A better alternative to the current clinical treatment would be to construct a scaffold matrix with natural or synthetic polymers, polymer blends, or polymer–ceramic composites, which could limit the need for donor tissue, extended surgery, and risk of infection [18]. Studied extensively, SF have favorable effects for bone tissue engineering offering advantages such as biodegradability, biocompatibility, mechanical behavior, ease of processability, exceptional flexibility, and porosity [19]. Mobini et al. introduced a regenerated silk-based composite material composed of 3D silk fibers embedded in a porous matrix of fibroin as reinforcement [20]. This porous scaffold, prepared by freeze-drying, showed enhanced mechanical strength to withstand compressive forces [20]. The compressive moduli and stress significantly increase due to the reinforcing nature of the fibers [20]. In addition to mechanical strength, the porous scaffolds supported attachment, proliferation, and differentiation of human mesenchymal stem cells (hMSCs) (Figure 3) [20]. Considering the enhanced mechanical and biological properties, the composite scaffolds appear to be an interesting hybrid structure in bone tissue engineering. 

To obtain a porous scaffold with uncompromised mechanical strength is quite challenging. Initially to understand the effects of scaffold architecture and biomechanics, Correia et al. optimized and modeled silk scaffolds in terms of architecture (porosity, pore dimensions, and pore geometry) and stiffness [21]. The group seeded scaffolds with human adipose-derived stem cells (hASCs) to evaluate cell proliferation and differentiation, matrix production, and calcification [21]. The porous SF scaffolds were made using the conventional freeze-drying technique and salt-leaching method with different solvents to obtain various pore sizes and structures (lamellar and spherical pores) [21]. To accelerate bone formation, it was determined that 400–600 μm porous lamellar scaffold was the optimal bone tissue formation due to increased adipose activity, equilibrium modulus, and calcium deposition [21]. Thus, this optimization study signified the importance of appropriate scaffold design in bone tissue applications.

As mentioned earlier, porous 3D silk scaffolds enhanced biodegradability and biocompatibility. Taking an osteoconductive approach, Bhumiratana et al. investigated the effect of hydroxyapatite (HA) mineral with hMSCs in a porous silk scaffold [22]. The group also incorporated HA microparticles into the silk sponge matrix and seeded them with MSCs [22]. When compared to pure SF, the highly osteogenic composite scaffolds showed enhanced differentiation and rapid bone formation due to the faster recruitment of cells [22]. The osteoconductivity of the HA provides a starting place for the nucleation of new minerals, which increases bone matrix production [22]. The embedded HA mineral enhances the post-mechanical properties of the porous silk scaffold after bone formation, making it a suitable candidate for bone tissue regeneration.

Many attempts have also been made to mimic the natural inorganic composition of bone through artificial means. These osteoconductive/inductive bioceramic materials, such as calcium phosphates, calcium sulfates, and silica, are widely used for bone repair and substitute [23]. To meet the requirements for complex bone repair, Xie et al. integrated SF into a bioceramic, calcium polyphosphate (CPP) scaffold crosslinked with glutaraldehyde [23]. The group reported enhanced compressible strength and reduced material degradation from the incorporation of SF into the CPP structure [23]. The SF/CPP bioceramic scaffold also exhibited improved biocompatibility and lowered cellular toxicity than those of the CPP and HA alone [23]. To repair or regenerate large bone defects, bioactive three-dimensional (3D) scaffolds can play a key role. Xu et al. fabricated porous hierarchical bioceramic–SF (BC–SF) composite scaffolds with a combination of 3D-plotting and freeze-drying. The study indicates improved in vivo osteogenesis of bone marrow stromal cells (BMSCs) in the layer by layer scaffold. The porous BC–SF scaffolds showed excellent apatite-mineralization and mechanical strength. Compared to BC scaffolds, BC–silk scaffolds also showed enhanced cell attachment rate, proliferation, ALP activity, and bone-related gene expression [24]. With the combined advantages of polymer and ceramic composites, scaffolds can overcome the brittle nature of ceramics only, making it a suitable material for bone tissue engineering.

To regenerate bone tissue, it is necessary to provide a scaffold that bears structural resemblance to natural ECM in bone. For ECM-like structures, Park et al. developed a 3D electrospun silk fibroin (ESF) scaffold with alterable pore size using a salt-leaching method. In the study, ESF scaffolds were compared with commercially available porous 3D polylactic acid (PLA) scaffolds. Results showed higher proliferation of osteoblasts and bone formation activities in vitro and in vivo [25]. In another study, Shao et al. fabricated an HA–SF fibrous core encase SF shell using coaxial electrospinning in an aqueous solvent to mimic the parallel collagen fibrils in native bone [26]. With aligned nanofibers, the scaffold showed improved mechanical properties, with a 90-fold higher initial modulus and a 2-fold breaking stress, compared to that of randomly oriented scaffold [26]. Osteoblasts attained from the differentiation of MSC were seeded onto HA–SF and a pure SF scaffold to assess the suitability for bone tissue engineering [26]. The results proved that the composite nanostructured fibers possessed higher biocompatibility, biomineralization, cell adhesion, and proliferation in comparison to the control [26]. This physical blending technique showed encouraging results for use in bone tissue applications. Another promising ECM for better bone formation would be the inclusion of non-mulberry silk fibroin (NSF) from the tropical tasar silkworm into PCL nanofibers. To study the effectiveness, Bhattacharjee et al. investigated the matrices of blended and grafted NSF into and onto electrospun PCL substrates, as an in vivo implant over 60 days [27]. At the bone defect site in the distal metaphysis region of the rabbits’ femur, the implant showed early bone formation, infiltration, and strong bonding at the bone–implant interface. Bioactive polymers such as NSF grafted with PCL nanofibrous matrix and nano-hydroxyapatite and loaded with bone morphogenic protein-2 (rhBMP-2) and transforming growth factor beta (TGF-b) have an edge over mulberry SF [28]. The aiding bioactivity, cell viability, and osteoinductivity with the already enhanced mechanical properties of SF make non-mulberry SF scaffold another worthy candidate for bone tissue engineering.

### 3.2. Silk for Eye Regeneration

SF has applications in corneal tissue engineering due to its biological properties. The main problem with corneal transplantation is the biocompatibility and stability of the artificial cornea tissue after penetrating keratoplasty. The grafts for keratoplasty are often taken from donated corneal tissue or replaced with the heterogeneous forming material [29]. Amniotic membrane (AM) transplantation has long been used in the field of ophthalmology as a tissue bandage for cornea infections and substrate for ocular surface repair [30]. It has become very popular recently with the advancement of regenerative medicine and tissue preservation techniques. Since AM comes from the innermost layer of the placenta, it can function in the eye as a basement membrane substitute with growth factors that promote epithelial healing on the surface of the eye. However, infectious disease transmissions, such as human immunodeficiency virus (HIV), hepatitis B virus, hepatitis C virus, and syphilis are potential risks [31]. To overcome transmission and shortages of donor corneas, SF materials that could generate minimal immune and inflammatory responses were used to support corneal epithelial cells’ adherence, proliferation, and differentiation, as well as the growth of human corneal endothelium with a substratum suitable for transplant. Madden et al. prepared 5 μm thick SF transparent films modified with coatings of collagen IV, FNC Coating Mix™, and a chondroitin sulphatelaminin mixture to assess cell attachment and proliferation [32]. The group achieved successful cell confluency and proliferation with the collagen coating, which makes it a potential substratum for the transplantation of tissue-constructs for keratoplasty. The reason for selecting SF membranes/scaffolds for corneal tissue regeneration is due to its potential benefits, including biocompatibility, mechanical integrity, transparency, and slow biodegradation [33]. To meet these functional requirements, SF and its complexes must replicate the corneal stromal tissue architecture and modulate the activities of the epithelial cells. Lawrence et al. have designed and characterized silk film processing techniques to mimic the 2 mm thick corneal collagen lamellae layer [33]. The group introduced 0.5–5.0 mm diameter pores and surface patterns into silk films to show the utility for cell alignment and trans-lamellar diffusion of nutrients [33]. By fabricating silk film constructs that resemble the physiological characteristics of a normal cornea, serious complications can be reduced after implantation. Currently, there is broad agreement that the long-term stability and usability of artificial cornea need to possess mechanical properties of the normal cornea, such as microporosity and optical clarity; the ability to support cornea cells continuously is also critical to prevent the destruction of collagenase and to promote corneal tissue regeneration [34].

**Figure 4:**
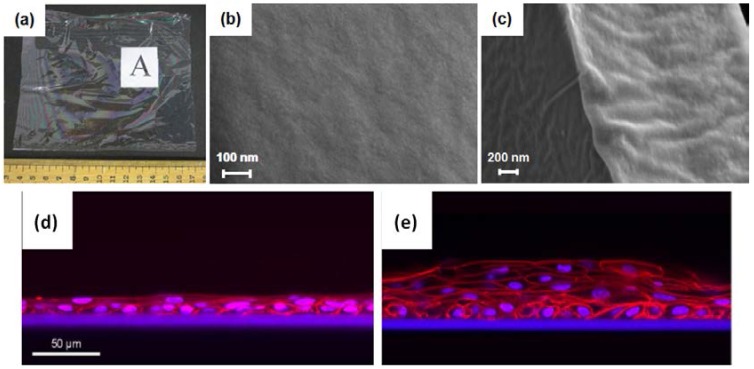
(**a**–**c**) SEM images of *B. mori* silk fibroin membranes for eye regeneration. Histology by confocal microscopy after cultivation of primary human corneal limbal epithelial cells for 12 days on SF membranes; (**d**) without feeder cells and (**e**) co-cultured with feeder cells (irradiated 3T3 murine fibroblasts). (Reproduced with permission from [32].)

To gain insight into how cell responses are modulated, SF film properties can be modified to test for attachment and proliferation of corneal epithelial cells. The tissue-engineered films can be composed of SF and other synthetic biopolymers (with/without biological cues). Suzuki et al. mixed SF solution with poly(ethylene glycol) (PEG) to increase the permeability and topographic roughness of the surface (Figure 4). The increased rigidity resulted in only a slight change of the cell proliferation in the long term [35]. Since the PEG-treated substratum did not improve the attachment and proliferation of cells, making it a less viable choice than the non-treated membranes. While the tailored silk film substrates did contribute to tissue development, showing a higher nuclear-to-cytoplasmic ratio and suggesting a more proliferative phenotype [35]. In other studies, the functionalization of arginine-glycine-aspartic acid (RGD)-containing peptide onto SF scaffolds/substrates showed increases in cells attachment. But when Bray et al. conjugated RGD with SF at different ratios, no enhanced human corneal limbal epithelial cells attachment could be seen with the presence of RGD sequences. Thus, the group concluded that the RGD ligands may have a more complex mechanism [35]. Even though RGD SF fails to improve adhesion and growth, silk fibroin derived from the wild silkworm *Antheraea pernyi* (APSF) with a natural RGD sequence is still a promising alternative to *Bombyx mori* SF. A very recent study by Hazra et al. has shown that APSF from non-mulberry provides suitable and similar support, attachment, migration, growth, and support of epithelial cells and keratocytes in rabbit eyes following implantation. This study sought to evaluate the potential of non-mulberry silks to be used as a matrix for corneal reconstruction and growth of new corneal epithelial cell sheets and limbal stem cell with the expression of cytokeratin 3, vimentin, and ABCG2 [36]. Further studies are needed to provide a complete characterization of SF biomaterials for future application in corneal tissue engineering.

Ocular drug delivery has been a major challenge due to the unique anatomy and protective mechanisms of the eye, which result in low bioavailability of drugs through the various ocular tissues [37]. Novel drug delivery strategies such as bioadhesive materials is an efficient solution to improve and sustain ocular drug therapeutic efficacy at the target site [37]. Dong et al. formulated SF-coated liposomes loaded with ibuprofen to demonstrate sustained ocular drug delivery [38]. Using silk fibroin as a vehicle for delivery, cellular adhesion and cytotoxicity assay were tested on human corneal epithelial cells [38]. The group showed sustained drug release and permeation of ibuprofen with tunability, making it a promising ophthalmic drug delivery system and regenerative medicine.

### 3.3. Silk for Nerve Regeneration

**Figure 5:**
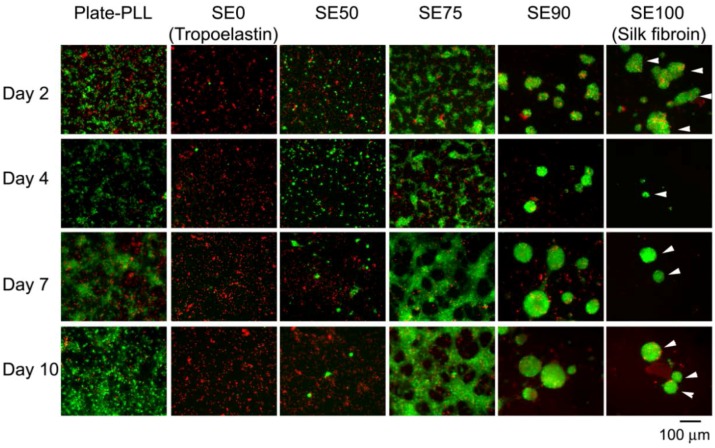
Fluorescence images of cell viability assay of neuronal cultures on silk-tropoelastin films (SE0 (pure tropoelastin), SE50, SE75, SE90, SE100 (pure silk)) in comparison to control (Plate-PLL) at different mixing ratios over days in vitro (green, live cells; red, dead cells). Reproduced with permission from [42], Copyright Wiley Inc., 2013.

Debilitating brain and spinal injuries, degenerative neurological diseases, and peripheral nerve damage have remained a clinical challenge. For peripheral nerve repair, the typical choices are the direct suturing of nerve ends, the implantation of a nerve autograft, or the electrical stimulation of neurites to bridge the nerve gaps and facilitate nerve regeneration. However, these techniques are limited and hampered by tissue availability, donor-site morbidity, and scar tissue formation insulating the electrodes [39]. To promote the guided regeneration of nerve cells, SF is a promising biofunctional material interface for nerve repair with remarkable biocompatibility, biodegradability, dielectric properties, and mechanical flexibility. To address the effect of pure SF films on the cell, Benfenati et al. cultured dorsal root ganglion (DRG) neurons onto bare SF films in vitro. The group demonstrated SF films alone can support the growth and neurite extension of DRG, which in turn can promote the repair of the peripheral nervous system [40]. Through in vivo studies, the group demonstrated that SF films preserve the firing and retention of electrophysiological properties, such as the intracellular free Ca^2+^ concentration [40]. The group also demonstrated that SF films functionalized with nerve growth factor (NGF) can enhance and promote the adhesion, migration, and proliferation of neurites. To exploit the biologically favorable qualities of silk-based composite materials, Gu et al. prepared a hybrid nerve scaffold by modifying SF/chitosan blend materials with nerve-cell-derived ECM. The addition of natural ECM scaffolds creates an ideal microenvironment for the nerve cell to attach, differentiate and grow. In the end, the group was able to bridge a 10 mm long sciatic nerve gap in rats [41]. Also, the results from morphological and electrophysiological analysis by the hybrid scaffold showed the regenerative capability to be greater than a plain chitosan/SF scaffold only and on par with an acellular nerve graft [41]. Unlike acellular nerve graft, a tissue-derived ECM scaffold, the SF-derived ECM-modified scaffold derived from cell ECM does not suffer from tissue scarcity, pathogen transfer, and uncontrollable degradation kinetics, which represents a new alternative for supporting nerve regeneration [41]. Composites such as silk fibroin protein and recombinant human tropoelastin, based on charge, are important for constructing ECM of human nerve tissues with control of selective interactions of cell surface integrins with surface material charges and elasticity. Molecular interaction mechanisms can generate multifunctional silk/elastin protein alloys with tunable net charges that can regulate the growth and formation of charge-sensitive neuron networks (Figure 5) [42].

**Figure 6:**
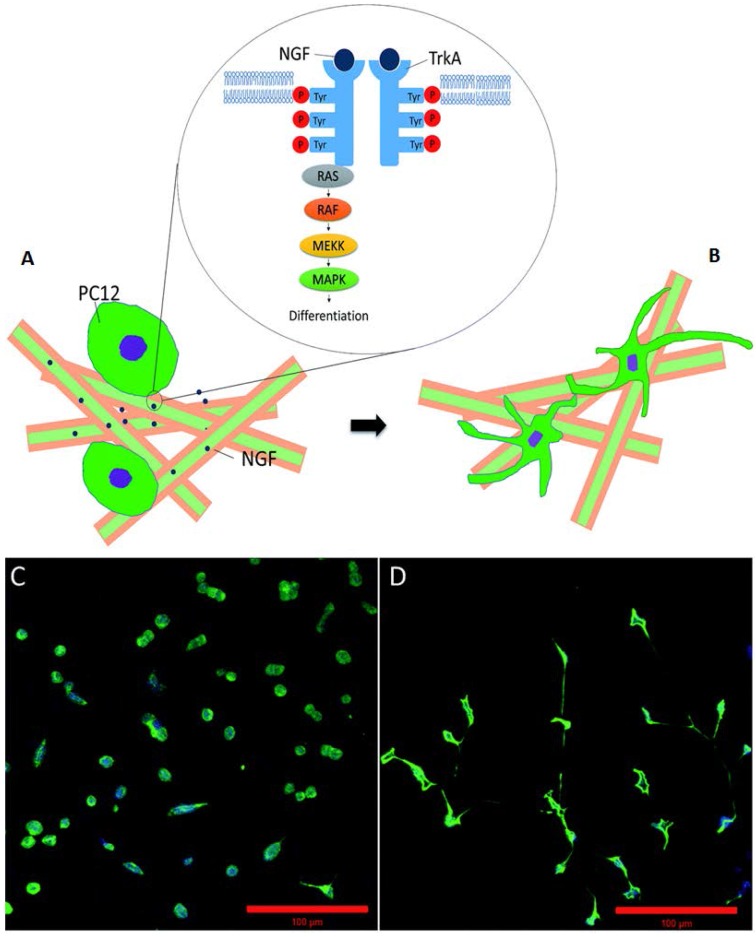
(**A**,**B**) Schematic illustration of the neuronal differentiation of PC12 cells on electrospun, plasma-treated poly(lactic acid)/silk fibroin/nerve growth factor (p-PS/N) scaffolds; (**C**,**D**) Expression of the neuronal protein (NF200) on different scaffolds: the advantage of p-PS/N (**D**) scaffolds over PS/N scaffolds without plasma treatment (**C**) is to support the differentiation of PC12 cells after 10 days. Reproduced with permission from [43], Copyright The Royal Society of Chemistry, 2015.

For protein drug encapsulation and controlled release, Tian et al. developed and fabricated a nanofiber composite comprising of a SF/NGF core coated with poly(lactic acid) using a coaxial electrospinning method to preserve the activity of the biomolecules (Figure 6) [43]. This process encapsulates nerve growth factor (NGF), or other drugs of interest, inside the SF scaffold, which allows for the preservation and sustained release of NGF [43]. Once NGF contacted the PC12 cells, it bound to TrkA, which activated the autophosphorylation of the receptor, and initiated the neuronal differentiation. A number of signaling cascades were involved during this process, such as the Raf/MEK/MAP kinase pathway and the PLCγ/PKC pathway [43]. The group showed that the PLA/SF/NGF scaffold enhanced attachment and differentiation of PC12 cells with elongated neurites outgrowth of 95 mm after 11 days. Air plasma treatment was also applied to PLA/SF/NGF scaffold to increase the surface hydrophilicity of the composite as well [43]. The plasma surface modification of polymers alters the hydroxyl group and the positive surface charge of SF, which improves neuronal stem cell affinity and enhances peripheral nerve regeneration [43].

Using co-axial electrospraying and electrospinning, Zhang et al. were able to blend a tri-composite nanofiber comprising of lysine-doped polypyrrole (PPy), regenerated spider silk protein (RSSP), poly(L-lactic) acid (PLLA), and NGF to draw different materials into a texture similar to that of the surrounding connective tissue [44]. By applying the two methods simultaneously, a core-shell structure with nanofiber branches can be created. This geometry also showed enhanced cell adhesion with pheochromocytomaPC12 cells and stable electrical and mechanical properties [44]. To promote Schwann cell outgrowth and axonal regeneration, the group applied electrical stimulation to the conductive composite nanofibers [44]. With the electrical stimulation enhancing the expression and the addition of NGF in cells, the scaffold was effective at repairing a 2.0 cm sciatic nerve gap in adult rats within 10 months with the slow leaching of NGF from the degradation rate of the tri-polymer scaffold [44]. The slow biodegradable of SF has the potential to stimulate and control peripheral nerve repair.

Few studies indicated the application of recombinant DNA technology in SF scaffolds in repairing peripheral neurons and nerve defects. Alternatively to SF coated with poly-D-lysine, White et al. also blended SF with recombinant human tropoelastin protein [45]. SF-tropoelastin film is a promising biomaterial for neural repairs with the additional benefit of being transparent and flexible. The group observed a 2.4-fold increase in neurite extension demonstrating that the SF-tropoelastin film supported peripheral neurons and Schwann cell growth [45]. Also, the group patterned the surface of the film with guided grooves to provide a biomaterial template for Schwann cell growth. With the addition of direction and alignment patterned, a Schwann cell was easily recruited to the site, improving the efficacy of nerve repair [45].

### 3.4. Silk for Skin Regeneration

SF have been increasingly investigated as a skin substitute material in tissue engineering due to its properties of biocompatibility, slow degradability, and hemostasis, which stimulate the collagen synthesis of fibroblasts and accelerate tissue reconstruction. For the formation of new tissue and organ, Sionkowska et al. prepared porous SF/collagen scaffolds obtained through lyophilization. This process decreases the tensile strength but increases the Young’s modulus. The temporary support of the composite matrix allows for growth and viability of the cells in tissue engineering [46]. For the treatment of burn wounds, Vasconcelos et al. successfully produced silk/elastin scaffolds, through lyophilization, which emulated the dermal extracellular matrix (ECM) [47]. With the combined elasticity of elastin and the already excellent biocompatibility and chemical stability of SF, accelerated re-epithelialization could be seen at the wound sites [47]. Furthermore, with the crosslinking of genipin, the group was able to reduce the pore size in the scaffold to mimic natural dermal constructs [47]. In another report, to mimic the epidermal structure, Bhardwaj el al. successfully fabricated porous 3D SF–keratin scaffolds obtained through freeze-drying with high porosity and swelling ability. The SF–keratin composite showed enhanced fibroblast growth, attachment, and proliferation along ECM deposition of collagen type I through the immunohistochemical expression [48].

**Figure 7:**
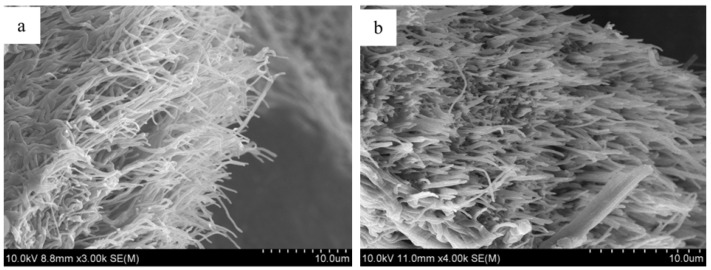
Cross-section images of electrospun SF fibers mat for skin reconstruction (**a**) before and (**b**) after cell culture for three days. Reproduced with permission from [50], Copyright Wiley Inc., 2013.

Even though traditional salt-leaching and electrospinning can offer a useful therapeutic option for the treatment of skin damage, such as burns, cells have a difficult time infiltrating the nanofibers due to their small pore size. To overcome this limitation, Sheikh et al. introduced cold-plate electrospinning, which allows for high porosity and facial shaping of the scaffold [49]. The controlled porosity and full-thickness 3D of the nanofibers lead to improved cell infiltration and skin reconstruction. Developing surface modification techniques for accelerated wound healing is of keen interest in biomedical applications. In another study, Chutipakdeevong et al. developed an electrospun SF nanofiber material blended with low molecular weight poly(ethylene oxide) (PEO) (Figure 7). After PEO extraction, through washing, the SF mat surfaces were functionalized with fibronectin, using carbodiimide chemistry, to induce cellular adhesion and cell migration [50]. Overall, the surface-modified SF mats showed good attachment, proliferation, and migration of normal human dermal fibroblasts (NHDF) [50].

**Figure 8:**
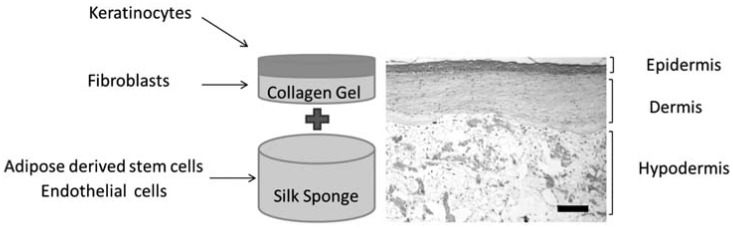
Experimental outline for a tri-layer culture in silk–collagen composite materials to mimic the skin structure. Reproduced with permission from [52], Copyright Wiley Inc., 2012.

Various novel techniques have also been developed to process SF into scaffolds for skin wound healing. By applying a foaming method, Sharma et al. developed the first microporous ternary composite scaffold consisting of chitosan, alginate, gelatin, and silk fibroins. These natural polymers have been shown individually to mimic the ECM of the body in other studies, supporting the growth and proliferation of cells. The addition of SF has been shown to strengthen the overall polymer structure mechanically and to slow down degradation. This offers time for the infiltration and formation of the surrounding tissue and ECM during tissue regeneration [51]. To create a sustainable 3D skin substitute and to identify optimal conditions, a full-thickness skin model should include not only an epidermis and dermis, but also a hypodermis layer. Bellas et al. constructed a tri-layer skin equivalent using SF sponge as the hypodermis, collagen as the dermis, and keratinocytes as the overlaying of the epidermis (Figure 8) [52]. The group seeded endothelial and adipose-derived stem cells onto the silk sponge and fibroblasts within the collagen gel layer [52]. The prepared full-thickness skin equivalent showed typical keratin 10 and collagens I and IV expressions with the production of glycerol and leptin, markers of adipose metabolism [52]. These membranes serve as a base for understanding factors, such as vasculogenesis, needed for a stable, functional model of skin for healing deep burn and incision wounds.

### 3.5. Silk for Tendon and Ligament Regeneration

Tendon and ligaments are hypocellular connective tissues responsible for joint movements, stress transfer, and joint stability [53]. They are regularly subjected to high physiological loads, thus commonly injured. To properly heal after injury, many factors need to be considered such as its anatomical location, vascularity, and the amount of tissue loss [54]. Surgical treatment with tendon grafts is often required, but is often complicated with reduced strength, stiffness, ruptures, and donor-site morbidity [55]. For more than 10 years, acellular dermal membrane (ADM) has been successfully used in the augmentation of ligament and tendon repairs. Biologic grafts processed from acellular human dermis can provide support and cellular regeneration for ruptured tendons with the preservation of the collagen structure found in the dermis [56]. The aim of tendon replacement is to fabricate a cell-scaffold construct with similar mechanical and functional characteristics as the native tissue in vitro for transplantation, and incorporate self-regeneration techniques at the injury site in situ to prevent these complications [53]. Considering the anatomy and physiology of native tendon tissues, Chen et al. formed a sponge scaffold by crossing collagen and SF fibers to provide good mechanical strength and enhanced cell attachment. Mimicking the native aligned collagen fibrous bundles, hESC-derived mesenchymal stem cells (hESC-MSCs) were also incorporated into the construct. The combination displayed improved cell alignment, attachment over larger collagen fibers, and tenocyte-like morphology under mechanical stimulus [53]. Tendon-related regeneration was also studied in a rat Achilles tendon injury model using SF/collagen composite fibers. Collagen type I & III, Epha4, and Scleraxis gene markers were expressed in hESC containing SF/Collagen composite fibers, showing effective cell–scaffold (matrix) interaction in the tissue regeneration cascade [53]. To examine the environment-modifying effect on the implantation site in situ, extracellular matrix (ECM) expression assays were performed by the group, showing increased ECM production. Since high tensile strength is an important criterion to meet when designing silk-scaffold-based constructs, the group also knitted a silk–collagen sponge scaffold, in another study, to further promote tendon regeneration [57]. To enable the migration and homing of stem cells into the scaffold, Shen et al. developed a bioactive knitted silk–collagen sponge scaffold by incorporating exogenous SDF-1 alpha, a cytokine. From the rat Achilles tendon injury models, SDF-1 alpha was shown to reduce inflammatory cell recruitment and enable stem cell migration into the scaffold, making it a practical application for tendon tissue [58].

To provide additional strength and support, hybrid fibrous scaffolds comprising both microfibers and nanofibers of SF with durable synthetic polymers can be used to enhance the tensile strength of the final materials. Sahoo et al. embedded and coated bioactive bFGF-releasing ultrafine PLGA fibers into slowly-degrading knitted microfibrous silk scaffolds. The biohybrid scaffold system acted as a favorable scaffold, enhancing cell attachment and proliferation, while the sustained release of bFGF mimicked the ECM in function, stimulating mesenchymal progenitor cell (MPC) proliferation and their tenogeneic differentiation [55]. The outcome of this study proved that porous SF containing microchannels and aligned microfibrous surfaces can promote the gene expression of tendon-specific ECM proteins and increase collagen production, resulting in bundled growth of tenocytes, which further enhances the deposition of collagen [55]. Thus, the architecture of the scaffold plays a significant role in generating an analog that has the potential to be used to repair injured ligaments and tendons.

Electrospun nanofibers are shown to have high tensile strength. Aiming to fabricate a hierarchical arrangement of collagen fibers in the native anterior cruciate ligament (ACL), Naghashzargar et al. developed a novel nano/micro-hybrid braid by electrospinning P3HB or PCL nanofibers onto a twisted SF cord. The braided cords have sufficiently high mechanical properties to withstand the cyclic loading experienced by ACL tissue in daily activities [59]. While tightly wrapped, the braided SF micro-filament was not influenced by the presence of the electrospun polymer nanofibers, which usually affects cellular in-growth and nutrient infiltration due to limited space available [59]. L929 fibroblasts were seeded onto the braided cords to test cytotoxic effects, cytocompatibility, cell viability, and extracellular matrix deposition [59]. With an increase of internal forces due to the interaction between nano- and micro-components, the cell viability on the new fibers is higher than that on uncoated SF fibers, indicating that the nano/micro-hybrid braided cords are promising for tendon and ligament tissue engineering. In other studies, the hydroxyapatite (HA) coating on SF fibers of a ligament scaffold was found to further improve mechanical strength and graft osseointegration with the host bone. By incorporating HA onto SF scaffolds, successful graft-to-host bone/ligament defects can be performed. He et al. soaked the SF sponge ends a number of times to deposit varying layers of HA. Since HA is hydrophobic, histological observations showed that HA-coated silk scaffolds were less favorable for initial cell attachments, but showed enhanced cell viability and sustained proliferation in silk graft for bone attachment [60]. From in vitro and in vivo tests, the group also showed osteogenic differentiation of bone marrow mesenchymal stem cells and improvements in osteoconductivity on the silk–HA scaffolds [60].

### 3.6. Silk for Cartilage Regeneration

Many patients suffer from cartilage degeneration due to genetic abnormalities, trauma, or osteoarthritis [61]. In severe cases, the patients will have various complications like pain and loss of extremity functions in their daily lives [61]. Articular cartilage is an avascular tissue, so a defect in the chondral area will propagate into a large area of the cartilage and finally require surgical intervention or replacement surgeries [61]. To resolve this problem, cartilage tissue engineering can be applied to control structural constructs and morphological features of cells and bioactive molecules that could interact with the tissue [61]. Artificial biomaterials or scaffolds exhibiting excellent characteristics, such as biocompatibility, biodegradability, and good cell affinity, could heal functional cartilage without scar tissue. To demonstrate the effect of the dependence of mechanical properties on chondrogenesis, Wang et al. prepared a composite of collagen and SF with poly-lactic-co-glycolic acid (PLGA) microsphere to obtain optimum conditions for cell proliferation at different collagen-to-silk fibroin ratios. Scaffolds containing a ratio of 7:3 collagen and silk fibroin provided optimal conditions [62]. The scaffold with PLGA microsphere incorporated was implanted surgically in osteochondral regions of rabbits [62]. Enhanced articular cartilage regeneration and integration could be seen by in vitro fluorescence staining of bone marrow stromal cells, indicating that collagen/silk fibroin composite scaffold can enhance the migration of pluripotent stem cells that differentiate into chondrocytes [62].

Jaipaew et al. developed a 3D porous silk fibroin (SF)/hyaluronic acid (HA) scaffold formed by freeze-drying. The scaffold was then seeded with human umbilical cord-derived mesenchymal stem cells (HUMSCs) and cultured in the chondrogenic medium [63]. The group reported sphere complexes of cells in the porous scaffold structure that express Col2a, Agg, and Sox9 markers for chondrogenesis, suggesting accelerated differentiation and functionality of the chondrocytes [63]. The unique 3D design in addition to the interconnected pores provided a high swelling ratio and enhanced water uptake, thereby, creating a soft and elastic characteristic mimicking the native cartilage. Bhardwaj et al. studied an SF/chitosan (CS) blended scaffold with bovine chondrocytes. SF served as a stimulating environment for cell adhesion and proliferation, while chitosan, having structural similarity with glycosaminoglycans (GAGs) and hyaluronic acid (HA), stimulates chondrogenesis in articular cartilage [64]. The cell scaffold was analyzed for chondrogenesis in vitro within two weeks through cell viability, histology, extracellular matrix components, content ofGAG and collagen types I and II analysis, and biomechanical property tests [64]. When compared to pure SF, this blended approach showed significant improvement of cell attachment, growth, and chondrogenic phenotype on the scaffolds. The SF/CS scaffold provided sufficient mechanical integrity compared to native tissue and also showed the highest GAGs and collagen formation in silk fibroin/chitosan (1:1) blended scaffolds [64]. Therefore, an ideal silk-based scaffold for cartilage regeneration should satisfy the needs for mechanical strength and structural resilience that a spatial structure would provide, as well as the cellular morphology and ECM composition of the native cartilage at the structural level. New silk-based composite scaffolds composed of multilayers with different thicknesses that can couple with chondrocyte-based connective tissues could be the best choice for cartilage regeneration in the future.

## 4. Conclusions

Tissue engineering is a fundamental approach to repair or replace portions of body tissues and organs that are damaged or non-functional. Silk fibroins are a natural biopolymer having numerous favorable structural properties and vast possibilities for chemical and mechanical modifications. Most notably, many applications take advantage of silk’s smooth texture, good biocompatibility/biodegradability, thermal stability, and excellent mechanical properties. The integration of these newly formed tissues with themselves and the surrounding natural environment is of great importance. Classic methods of joining tissue can concentrate mechanical stresses in the tissues as well as create sites for infection. Through surface modifications or compositing with other synthetic polymers, a novel silk polymer can be designed to set completely in human fluid environments and join connective tissues. Engineering silk materials to store a significant amount of elastic energy to perform mechanical work during their shape recovery can also be an avenue to explore. The key to developing novel silk biomaterials would be to figure out how to control the crystallization in SF when it is thermally treated or mechanically stretched. The fact that silk fibroins are a biocompatible natural protein provides silk materials with many advantages, such as minimal immune response, good adherence, and growth of cells on/in silk matrixes. With novel crosslinking biomaterial, silk materials could be designed to self-heal, resulting in new applications for tissue engineering where rapidly recover and load-bearing tissues are needed. 

True integration can be achieved by designing three-dimensional tissue formation made of biomaterials that not only act as temporary, artificial extracellular scaffolds for cells to adhere and form new tissue but also provide a biomatrix that support cues and signals to promote functional tissue connections. Among the various natural and synthetic polymers currently available, silk has shown remarkable promise in various medical/pharmaceutical fields, which will continually contribute to the exciting new developments in tissue engineering, drug delivery, biosensing, and fabrication technologies in the future.

## References

[1] Kundu B., Kurland N.E., Yadavalli V.K., Kundu S.C. (2014). Isolation and processing of silk proteins for biomedical applications. Int. J. Biol. Macromol..

[2] Kundu B., Kurland N.E., Bano S., Patra C., Engel F.B., Yadavalli V.K., Kundu S.C. (2014). Silk proteins for biomedical applications: Bioengineering perspectives. Prog. Polym. Sci..

[3] Koh L.-D., Cheng Y., Teng C.-P., Khin Y.-W., Loh X.-J., Tee S.-Y., Low M., Ye E., Yu H.-D., Zhang Y.-W. (2015). Structures, mechanical properties and applications of silk fibroin materials. Prog. Polym. Sci..

[4] Bhardwaj N., Rajkhowa R., Wang X., Devi D. (2015). Milled non-mulberry silk fibroin microparticles as biomaterial for biomedical applications. Int. J. Biol. Macromol..

[5] Talukdar S., Kundu S.C. (2012). A non-mulberry silk fibroin protein based 3D in vitro tumor model for evaluation of anticancer drug activity. Adv. Funct. Mater..

[6] Musson D.S., Naot D., Chhana A., Matthews B.G., McIntosh J.D., Lin S.T., Choi A.J., Callon K.E., Dunbar P.R., Lesage S. (2015). In vitro evaluation of a novel non-mulberry silk scaffold for use in tendon regeneration. Tissue Eng. Part A.

[7] Cao T.-T., Zhang Y.-Q. (2016). Processing and characterization of silk sericin from bombyx mori and its application in biomaterials and biomedicines. Mater. Sci. Eng. C.

[8] Mandal B.B., Kundu S.C. (2009). Osteogenic and adipogenic differentiation of rat bone marrow cells on non-mulberry and mulberry silk gland fibroin 3D scaffolds. Biomaterials.

[9] Cai K., Yao K., Lin S., Yang Z., Li X., Xie H., Qing T., Gao L. (2002). Poly(d, l-lactic acid) surfaces modified by silk fibroin: Effects on the culture of osteoblast in vitro. Biomaterials.

[10] Partlow B.P., Hanna C.W., Rnjak-Kovacina J., Moreau J.E., Applegate M.B., Burke K.A., Marelli B., Mitropoulos A.N., Omenetto F.G., Kaplan D.L. (2014). Highly tunable elastomeric silk biomaterials. Adv. Funct. Mater..

[11] Bhattacharjee P., Naskar D., Maiti T.K., Bhattacharya D., Kundu S.C. (2016). Non-mulberry silk fibroin grafted poly(ε-caprolactone) nanofibrous scaffolds mineralized by electrodeposition: An optimal delivery system for growth factors to enhance bone regeneration. RSC Adv..

[12] Cai Z.-X., Mo X.-M., Zhang K.-H., Fan L.-P., Yin A.-L., He C.-L., Wang H.-S. (2010). Fabrication of chitosan/silk fibroin composite nanofibers for wound-dressing applications. Int. J. Mol. Sci..

[13] Pillai C., Paul W., Sharma C.P. (2009). Chitin and chitosan polymers: Chemistry, solubility and fiber formation. Prog. Polym. Sci..

[14] Lamboni L., Gauthier M., Yang G., Wang Q. (2015). Silk sericin: A versatile material for tissue engineering and drug delivery. Biotechnol. Adv..

[15] Kundu B., Kundu S.C. (2012). Silk sericin/polyacrylamide in situ forming hydrogels for dermal reconstruction. Biomaterials.

[16] Aramwit P., Siritientong T., Kanokpanont S., Srichana T. (2010). Formulation and characterization of silk sericin–pva scaffold crosslinked with genipin. Int. J. Biol. Macromol..

[17] Wantanasiri P., Ratanavaraporn J., Yamdech R., Aramwit P. (2014). Fabrication of silk sericin/alginate microparticles by electrohydrodynamic spraying technique for the controlled release of silk sericin. J. Electrostat..

[18] Mottaghitalab F., Hosseinkhani H., Shokrgozar M.A., Mao C., Yang M., Farokhi M. (2015). Silk as a potential candidate for bone tissue engineering. J. Control. Release.

[19] Melke J., Midha S., Ghosh S., Ito K., Hofmann S. (2016). Silk fibroin as biomaterial for bone tissue engineering. Acta Biomater..

[20] Mobini S., Hoyer B., Solati-Hashjin M., Lode A., Nosoudi N., Samadikuchaksaraei A., Gelinsky M. (2013). Fabrication and characterization of regenerated silk scaffolds reinforced with natural silk fibers for bone tissue engineering. J. Biomed. Mater. Res. Part A.

[21] Correia C., Bhumiratana S., Yan L.-P., Oliveira A.L., Gimble J.M., Rockwood D., Kaplan D.L., Sousa R.A., Reis R.L., Vunjak-Novakovic G. (2012). Development of silk-based scaffolds for tissue engineering of bone from human adipose-derived stem cells. Acta Biomater..

[22] Bhumiratana S., Grayson W.L., Castaneda A., Rockwood D.N., Gil E.S., Kaplan D.L., Vunjak-Novakovic G. (2011). Nucleation and growth of mineralized bone matrix on silk-hydroxyapatite composite scaffolds. Biomaterials.

[23] Xie H., Gu Z., Li C., Franco C., Wang J., Li L., Meredith N., Ye Q., Wan C. (2016). A novel bioceramic scaffold integrating silk fibroin in calcium polyphosphate for bone tissue-engineering. Ceram. Int..

[24] Xu M., Li H., Zhai D., Chang J., Chen S., Wu C. (2015). Hierarchically porous nagelschmidtite bioceramic–silk scaffolds for bone tissue engineering. J. Mater. Chem. B.

[25] Park S.Y., Ki C.S., Park Y.H., Jung H.M., Woo K.M., Kim H.J. (2010). Electrospun silk fibroin scaffolds with macropores for bone regeneration: An in vitro and in vivo study. Tissue Eng. Part A.

[26] Shao W., He J., Sang F., Ding B., Chen L., Cui S., Li K., Han Q., Tan W. (2016). Coaxial electrospun aligned tussah silk fibroin nanostructured fiber scaffolds embedded with hydroxyapatite–tussah silk fibroin nanoparticles for bone tissue engineering. Mater. Sci. Eng. C.

[27] Bhattacharjee P., Naskar D., Maiti T.K., Bhattacharya D., Das P., Nandi S.K., Kundu S.C. (2016). Potential of non-mulberry silk protein fibroin blended and grafted poly (є-caprolactone) nanofibrous matrices for in vivo bone regeneration. Colloids Surf. B.

[28] Bhattacharjee P., Naskar D., Maiti T.K., Bhattacharya D., Kundu S.C. (2016). Non-mulberry silk fibroin grafted poly(є-caprolactone)/nano hydroxyapatite nanofibrous scaffold for dual growth factor delivery to promote bone regeneration. J. Colloid Interface Sci..

[29] Wang H.-Y., Wei R.-H., Zhao S.-Z. (2013). Evaluation of corneal cell growth on tissue engineering materials as artificial cornea scaffolds. Int. J. Ophthalmol..

[30] Meller D., Pauklin M., Thomasen H., Westekemper H., Steuhl K.-P. (2011). Amniotic membrane transplantation in the human eye. Dtsch. Arztebl. Int..

[31] Liu J., Lawrence B.D., Liu A., Schwab I.R., Oliveira L.A., Rosenblatt M.I. (2012). Silk fibroin as a biomaterial substrate for corneal epithelial cell sheet generationsf as a biomaterial substrate. Investig. Ophthalmol. Visual. Sci..

[32] Suzuki S., Dawson R.A., Chirila T.V., Shadforth A., Hogerheyde T.A., Edwards G.A., Harkin D.G. (2015). Treatment of silk fibroin with poly(ethylene glycol) for the enhancement of corneal epithelial cell growth. J. Funct. Biomater..

[33] Lawrence B.D., Marchant J.K., Pindrus M.A., Omenetto F.G., Kaplan D.L. (2009). Silk film biomaterials for cornea tissue engineering. Biomaterials.

[34] Madden P.W., Lai J.N., George K.A., Giovenco T., Harkin D.G., Chirila T.V. (2011). Human corneal endothelial cell growth on a silk fibroin membrane. Biomaterials.

[35] Bray L.J., Suzuki S., Harkin D.G., Chirila T.V. (2013). Incorporation of exogenous rgd peptide and inter-species blending as strategies for enhancing human corneal limbal epithelial cell growth on bombyx mori silk fibroin membranes. J. Funct. Biomater..

[36] Hazra S., Nandi S., Naskar D., Guha R., Chowdhury S., Pradhan N., Kundu S.C., Konar A. (2016). Non-mulberry silk fibroin biomaterial for corneal regeneration. Sci. Rep..

[37] Gaudana R., Ananthula H.K., Parenky A., Mitra A.K. (2010). Ocular drug delivery. AAPS J..

[38] Dong Y., Dong P., Huang D., Mei L., Xia Y., Wang Z., Pan X., Li G., Wu C. (2015). Fabrication and characterization of silk fibroin-coated liposomes for ocular drug delivery. Eur. J. Pharm. Biopharm..

[39] Yang Y., Chen X., Ding F., Zhang P., Liu J., Gu X. (2007). Biocompatibility evaluation of silk fibroin with peripheral nerve tissues and cells in vitro. Biomaterials.

[40] Benfenati V., Stahl K., Gomis-Perez C., Toffanin S., Sagnella A., Torp R., Kaplan D.L., Ruani G., Omenetto F.G., Zamboni R. (2012). Biofunctional silk/neuron interfaces. Adv. Funct. Mater..

[41] Gu Y., Zhu J., Xue C., Li Z., Ding F., Yang Y., Gu X. (2014). Chitosan/silk fibroin-based, schwann cell-derived extracellular matrix-modified scaffolds for bridging rat sciatic nerve gaps. Biomaterials.

[42] Hu X., Tang-Schomer M.D., Huang W., Xia X.X., Weiss A.S., Kaplan D.L. (2013). Charge-tunable autoclaved silk-tropoelastin protein alloys that control neuron cell responses. Adv. Funct. Mater..

[43] Tian L., Prabhakaran M.P., Hu J., Chen M., Besenbacher F., Ramakrishna S. (2015). Coaxial electrospun poly (lactic acid)/silk fibroin nanofibers incorporated with nerve growth factor support the differentiation of neuronal stem cells. RSC Adv..

[44] Zhang H., Wang K., Xing Y., Yu Q. (2015). Lysine-doped polypyrrole/spider silk protein/poly (L-lactic) acid containing nerve growth factor composite fibers for neural application. Mater. Sci. Eng. C.

[45] White J.D., Wang S., Weiss A.S., Kaplan D.L. (2015). Silk–tropoelastin protein films for nerve guidance. Acta Biomater..

[46] Sionkowska A., Lewandowska K., Michalska M., Walczak M. (2016). Characterization of silk fibroin 3D composites modified by collagen. J. Mol. Liq..

[47] Vasconcelos A., Gomes A.C., Cavaco-Paulo A. (2012). Novel silk fibroin/elastin wound dressings. Acta Biomater..

[48] Bhardwaj N., Sow W.T., Devi D., Ng K.W., Mandal B.B., Cho N.-J. (2015). Silk fibroin–keratin based 3D scaffolds as a dermal substitute for skin tissue engineering. Integr. Biol..

[49] Sheikh F.A., Ju H.W., Lee J.M., Moon B.M., Park H.J., Lee O.J., Kim J.-H., Kim D.-K., Park C.H. (2015). 3D electrospun silk fibroin nanofibers for fabrication of artificial skin. Nanomed. Nanotechnol. Biol. Med..

[50] Chutipakdeevong J., Ruktanonchai U.R., Supaphol P. (2013). Process optimization of electrospun silk fibroin fiber mat for accelerated wound healing. J. Appl. Polym. Sci..

[51] Sharma C., Dinda A.K., Potdar P.D., Mishra N.C. (2015). Fabrication of quaternary composite scaffold from silk fibroin, chitosan, gelatin, and alginate for skin regeneration. J. Appl. Polym. Sci..

[52] Bellas E., Seiberg M., Garlick J., Kaplan D.L. (2012). In vitro 3D full-thickness skin-equivalent tissue model using silk and collagen biomaterials. Macromol. Biosci..

[53] Chen J.L., Yin Z., Shen W.L., Chen X., Heng B.C., Zou X.H., Ouyang H.W. (2010). Efficacy of hESC-MSCs in knitted silk-collagen scaffold for tendon tissue engineering and their roles. Biomaterials.

[54] Anitha A., Sowmya S., Kumar P.S., Deepthi S., Chennazhi K., Ehrlich H., Tsurkan M., Jayakumar R. (2014). Chitin and chitosan in selected biomedical applications. Prog. Polym. Sci..

[55] Sahoo S., Toh S.L., Goh J.C. (2010). A bFGF-releasing silk/PLGA-based biohybrid scaffold for ligament/tendon tissue engineering using mesenchymal progenitor cells. Biomaterials.

[56] Blum B.E., Burgess A.V. (2009). Special segment: Soft tissue matrices—One form of acellular human dermis for use in tendon and ligament repairs in the foot and ankle. Foot Ankle Spec..

[57] Chen X., Yin Z., Chen J.-L., Liu H.-H., Shen W.-L., Fang Z., Zhu T., Ji J., Ouyang H.-W., Zou X.-H. (2014). Scleraxis-overexpressed human embryonic stem cell–derived mesenchymal stem cells for tendon tissue engineering with knitted silk-collagen scaffold. Tissue Eng. Part A.

[58] Shen W., Chen X., Chen J., Yin Z., Heng B.C., Chen W., Ouyang H.-W. (2010). The effect of incorporation of exogenous stromal cell-derived factor-1 alpha within a knitted silk-collagen sponge scaffold on tendon regeneration. Biomaterials.

[59] Naghashzargar E., Farè S., Catto V., Bertoldi S., Semnani D., Karbasi S., Tanzi M.C. (2015). Nano/micro hybrid scaffold of PCL or P3HB nanofibers combined with silk fibroin for tendon and ligament tissue engineering. J. Appl. Biomater. Funct. Mater..

[60] He P., Sahoo S., Ng K.S., Chen K., Toh S.L., Goh J.C.H. (2013). Enhanced osteoinductivity and osteoconductivity through hydroxyapatite coating of silk-based tissue-engineered ligament scaffold. J. Biomed. Mater. Res. Part A.

[61] Tuli R., Li W.-J., Tuan R.S. (2003). Current state of cartilage tissue engineering. Arthritis Res. Ther..

[62] Wang J., Yang Q., Cheng N., Tao X., Zhang Z., Sun X., Zhang Q. (2016). Collagen/silk fibroin composite scaffold incorporated with plga microsphere for cartilage repair. Mater. Sci. Eng. C.

[63] Jiang J., Ai C., Zhan Z., Zhang P., Wan F., Chen J., Hao W., Wang Y., Yao J., Shao Z. (2015). Enhanced fibroblast cellular ligamentization process to polyethylene terepthalate artificial ligament by silk fibroin coating. Artif. Organs.

[64] Bhardwaj N., Nguyen Q.T., Chen A.C., Kaplan D.L., Sah R.L., Kundu S.C. (2011). Potential of 3-D tissue constructs engineered from bovine chondrocytes/silk fibroin-chitosan for in vitro cartilage tissue engineering. Biomaterials.

